# Diversity of high risk human papilloma viruses in women treated with antiretroviral and in healthy controls and discordance with cervical dysplasia in the South of Benin

**DOI:** 10.1186/s13027-016-0090-9

**Published:** 2016-08-15

**Authors:** Callinice D. Capo-chichi, Blanche Aguida, Nicodème W. Chabi, Jocelyn Acapko-Ezin, Jonas Sossah-Hiffo, Vidéhouénou K. Agossou, Toussain Anagbla, Marcel Zannou, Fabien Houngbé, Ambaliou Sanni

**Affiliations:** 1 Molecular Biomarkers in Cancer and Nutrition (BMCN), Unit of Biochemistry and Molecular Biology (UBBM), Institute of Biomedical Sciences and Applications (ISBA), Faculty of Sciences and Technologies (FAST), University Abomey-Calavi (UAC), 04BP488, Cotonou, Benin; 2 National University Hospital (CNHU-HKM), Cotonou, Benin; 3 Hospital Mènontin, BP 160 Cotonou, Benin

**Keywords:** HIV, ARV, HPV, lamin A/C deficiency, Cervical dysplasia

## Abstract

**Background:**

High risk oncologic Human Papilloma Virus (HPV) is one of the leading causes of cervical cancer worldwide. We investigated HPV genotypes among women living or not with Human Immuno-deficiency Virus (HIV) in two major hospitals in the south of the republic of BENIN in the city of Cotonou. Our objective is to investigate the association of high risk-HPV to cervical dysplasia among women under stringent anti-retroviral (ARV) treatment and in controls without HIV.

**Methods:**

The investigation was carried out within 1 year period in two groups of adult women: one group with HIV1 infection and under ARV therapy in the National University Hospital (CNHU-HKM) designated as CH group (*n* = 86); and one control group without HIV infection and attending the hospital Mènontin for routine gynecologic checkup and designated as ME group (*n* = 86). Cells derived from cervical uterine smears (CUS) were used for this investigation. The samples in ME group were selected to have similar lamin A/C profile with CH group. HPV genotypes were assessed by polymerase chain reaction (PCR) while lamin A/C expression profile was assessed by western blotting to corroborate the risk of cervical dysplasia.

**Results:**

HPV56 is dominant in CH group while HPV66 is dominant in ME group. 31 % of women in CH group are infected with HPV compared to 23 % in ME group. Quadruple and quintuple HPV infections are more observed among CH group but not in ME group making HPV counts of 43 in CH group and 27 in ME group. Cervical dysplasia are present in 5 % (4/86) of women in CH group and in 1 % (1/86) of women in ME group at the time of CUS collection. The adjustment of the risk to develop cervical cancer in the future related to HPV infection and the total loss of lamin A/C is not significantly different in both groups.

**Conclusion:**

Women living with HIV are more sensitive to multiple HPV infection but not all HPV infections generated cervical dysplasia. The effectiveness of antiretroviral therapy in CH group may reduce significantly the frequency of cervical dysplasia.

## Background

Human Papilloma Viruses (HPV) viruses come in diverse genotypes. High risk HPVs (HR-HPV) are some of the leading causes of cervical cancer worldwide [1–6]. Over 200 genotypes have been identified and the HR-HPVs are linked to the occurrence of cervical carcinomas [1–6]. Health disparities in developed and developing countries render some population such as African, Asian, Hispanic and Mexican women more vulnerable to these diseases [6]. The co-infection HPV/HIV is more investigated due to the collaborative effects of both viruses to generate cervical dysplasia [7, 8]. HPV infections are more observed in population living with HIV due to the deregulation of the immune system [9, 10]. Underneath the complex interaction between nuclear components and viral components toward cancer development, the stability of lamin A/C should always be taken into consideration [11, 12]. Although the high frequency of cervical dysplasia among women living with HIV was often associated to the co-infection HPV/HIV [1–8]; there are factors else than HPV that may also be involved in cervical dysplasia [11, 12]. Studies performed in four West African countries including Benin, has shown that cervical cancer ranks number one in women living with HIV and number second in women negative for HIV [13]. The present study was done to verify the HPV genotypes among women living with HIV and treated with ARV compared to women with no HIV infection and the association to the loss of lamin A/C and cervical dysplasia in the city of Cotonou (South of the Republic of BENIN).

## Methods

### Population study

The investigation was carried out within 1 year period in two groups of adult women: one group with HIV1 infection and under ARV therapy in the National University Hospital (CNHU-HKM), designated as CH group (*n* = 86); and one control group without HIV infection and attending the hospital Mènontin for gynecologic routine checkup and designated as ME group (*n* = 86). Women in group CH with HIV1 infection are under stringent anti-retroviral treatment at CNHU-HKM while women in group ME without HIV infection are not receiving any treatment. The CH group is under free stringent ARV treatment program offered by the Ministry of Health in BENIN.

The inclusion criteria are women of 20 to 60 years old (average 38 years) and sexually active. The exclusion criteria are women in menstruation or within third month post-partum. Signed Informed consent was obtained from all women before the collection of cervical uterine smears (CUS). Sample collections were carried out in the gynecological service under the supervision of a gynecologist and nurses. The Cervical Uterine junction was visualized with colposcopy before CUS collection [11, 12]. This study was approved by the Research Ethic Committee of the Institute of Biomedical Science and Applications (CER-ISBA), and by the Ministry of Health in Republic of BENIN. HIV status was determined in the referral hospital (CNHU-HKM) with a rapid HIV test (Determine^(R)^, Abbott Diagnostics) or Genie2^(R)^ (Bio-Rad, Marnes-La-Coquette, France) test as previously reported [13].

### Cell collection and processing

To collect CUS for HPV and lamin A/C analyses, a disposable sterile speculum was introduced in the vagina along with a disposable cytobrush to reach the cervical-uterine junction where cells were collected by rotating the cytobrush clockwise twice. The brush was placed in a 50 ml collection tube containing 5 ml of sterile ice cold phosphate buffer saline (PBS) to collect cells. Cells were kept on ice and delivered within an hour to the laboratory (Unit of Biochemistry and Molecular Biology UBBM, Cotonou, BENIN) for processing. All collection tubes were centrifuged to gather cell pellets which were washed once with ice cold PBS before splitting them in several Eppendorf tubes (i) for the extraction of genomic DNA to genotype HR-HPV; (ii) for the analysis of lamin A/C proteins by western blot. The procedures followed were in accordance with the ethical standards of our institutional committee on human experimentation and in accordance with the Helsinki Declaration as previously reported [12].

### Reagents

HPV multiplex primers, Agarose powder, Ethylenediaminetetraacetic acid (EDTA), 2- deoxyribonucleic acid (DNA) ladder and ethidium bromide were from Sigma-Aldrich (France). Tris-Base, glycine, sodium dodecyl sulfate, bis-acrylamide and nitrocellulose membrane, the protein ladder and the peroxidase conjugated secondary antibodies anti-rabbit and anti-mouse were purchased from Bio-Rad Inc (USA). Sodium chloride (NaCl), potassium chloride (KCl), Tween-20, protease inhibitor phenyl-methyl-sulfonyl fluoride (PMSF), 2-mercaptoethanol, methanol, glycerol, 1,4-Dithiothreitol (DTT), sodium fluoride (NaF), sodium azide (NaN_3_), Tris-Hydrochloride (tris-HCl) and sodium dodecyl sulfate (SDS, NaC_12_H_25_SO_4_) were from Sigma-Aldrich (USA). The primary antibody against lamin A/C was purchased from Transduction Lab (USA). Antibody against ß-tubulin was from Santa Cruz Biotechnology (CA, USA). The chemo-luminescence reagent “Super Signal West Dura Extended Duration Substrate” made by PIERCE was from Thermo Scientific (Rockford, IL USA) and was used on Western blot membranes for protein revelation after exposure to X-ray films [12].

### DNA extraction

DNA was extracted from cell pellets (i) by the phenol-chloroform method after cell membrane disruption with lysis buffer containing proteinase K (20 mg/ml) and RNAse as previously reported [12]. Phenol was added to the cell lysate (V/V) and centrifuged at 10,000 rpm at 4 °C for 10 min to collect the upper aqueous phase into a new Eppendorf tube followed by the addition of chloroform (V/V) and another centrifugation (10,000 rpm at 4 °C for 10 min). The supernatant was collected in a new Eppendorf tube and ice cold ethanol (96 %) was added at -20 °C for 4 h to precipitate DNA. The DNA pellet was recovered after centrifugation (12,000 rpm at 4 °C for 10 min), was washed with ice cold ethanol (70 %) and then collected after centrifugation at 12,000 rpm at 4 °C for 5 min. The DNA pellet was air dried at 55 °C for 20 min, and solubilized in tris-EDTA (TE) buffer. The concentration of DNA was measured with a spectrophotometer (260 nm). Soluble DNA extract is stored in freezer until needed for genotyping of HPV by Polymerase chain reaction “PCR” [12].

### PCR and genotyping of HPV

The quality of DNA was assessed by amplifying ß-actin gene before setting up the PCR with HPV multiplex primers for genotyping. A nested multiplex PCR (NMPCR) assay that combines degenerate E6/E7 consensus primers and type specific primers was done as previously reported [12]. For each PCR reaction, positive and negative controls were used. A first consensus PCR was performed with human sequences (GPEG from SIGMA-Aldrich) to confirm the presence of HPV DNA. After the first PCR the amplified product was used again for a second amplification with the set of multiplex primers (nested PCR) to determine the genotype of HPV as previously described [12]. The multiplex primers used can detect the high grade oncogenic HPV and the low grade oncogenic HPV. The genotypes were divided in 4 groups according to the set of primers. The set 1 of primers is able to detect HPV16; HPV 18; HPV 31; HPV 59; HPV 45 and yield PCR products of 457; 322; 263; 215 and 151 base pairs (bp), respectively. The set 2 of primers is able to detect HPV 33; HPV 6/11; HPV 58; HPV 52; HPV 56; and yield PCR products of 398; 333; 274; 229 and 181 bp respectively. The set 3 of primers is able to detect HPV35; HPV 42; HPV 43; HPV 44 with PCR products of 358; 277; 219 and163 bp respectively. The set 4 of primers is able to detect HPV68; HPV 39; HPV 51; HPV 66; yielding PCR products of 333; 280; 223 and 172 bp respectively. The PCR products were run through 1.5 % agarose gel to separate amplicons according to their size. Amplified bands were detected by ethidium bromide staining under UV light. The HPV genotype was determined according to the molecular size of the band as specified previously [12].

### Cell lysates and lamin A/C analysis

Cell pellets (ii) were put in lysis buffer A [50 mM tris-HCl (pH 7.9), 150 mM NaCl, 0.1 mM EDTA, 1 % NP-40, 0.5 mM DTT, 0.5 mM PMSF, 30 mM NaF and 0.5 % protease inhibitor cocktail] and kept on ice for 30 min with vigorous agitation every 5 min as previously described [12]. Samples were boiled in SDS sample buffer for 5 min and store in freezer until analysis. Before western blotting protein samples were boiled again, loaded on 7.5 % SDS-polyacrylamide gels and run at 100 volts for 2 h in tris-glycine buffer, followed by a transfer to nitrocellulose membranes with transfer buffer containing tris-glycine and 20 % methanol. The membranes were blocked with 5 % milk in 1X Tris-buffered-saline (TBS) containing 0.1 % Tween-20 (TBST) for 30 min at room temperature before incubation in primary antibody rabbit-anti-lamin A/C at room temperature. The blots were washed 4 times for 10 min with TBST and incubated with HRP-conjugated secondary antibody anti-rabbit. The blots were washed 4 times for 15 min with TBST and incubated for 3 min in Super Signal West Dura Extended Duration Substrate before exposure to x-ray film and a film developer for the detection lamin A/C or of ß-tubulin as a loading control [12].

### Statistical analyses

This study is a transversal case control study between HIV infected group (CH) and non HIV group (ME). The Fisher test (F-test) was used on Excel software to compare the prevalence of HPV infection between groups CH and ME. The CUS with HPV are given the value of 0 if there are not infected with HR-HPV, the value of 1 if they are infected with one type of oncogenic HPV, the value of 2 if they are co-infected with two types of HR-HPV, the value of 3 if they are co-infected with three types of HR-HPV, the value of 4 when are co-infected with 4 types of HR-HPV and the value of 5 when they are co-infected with five types of HR- HPV; the difference between group CH and ME is considered significant when *p <0.05.*

The non-parametric Mann-Whitney test was used to compare lamin A/C profile between both groups. Lamin A/C profiles were given the value of 3 when normal expression was observed, the value of 2 for weak expression of lamin A/C and the value of 0 for total absence of lamin A/C. The difference between group CH and ME groups is considered significant when *p <0.05.*

### Video colposcopy

Women uterine cervix was examined with a video Colposcopy (SONY) by a gynecologist. Staining with dilute acetic acid followed by Lugol’s iodine solution was done to reveal the cervical lesions if present. The lesions of the cervix appeared white when stained with acetic acid (visual inspection with acetic acid, VIAA+) and yellow when stained with Lugol’s iodine solution (visual inspection with Lugol’s solution, VILI) as previously reported [11, 12].

### Ethical considerations

The protocol of this study including objectives, participants, sample collection, analysis, and reporting of data was approved by the Ethics Committee of the Institute of Biomedical Sciences and Applications (CER-ISBA) and by the Ministry of Health in Republic of BENIN. The objectives of the study were explained to all participants before inclusion. Signed informed consent was freely obtained from all participants before sample collections for the study [12].

## Results

### HR-HPV status in HIV population (CH group)

**Fig. 1:**
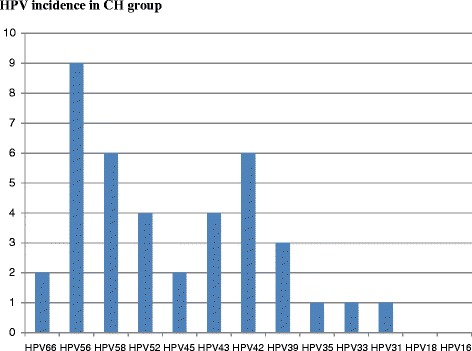
Incidence of HPV types in HIV infected women in group CH. Genotype HPV56 is more frequent than HPV 58, HPV 42 followed by HPV52, HPV43; HPV56, HPV39; HPV45, HPV66 and HPV35, HPV33, HPV31. HPV18 and HPV16 were not detected in this CH group

Dual, quadruple and quintuple HR-HPV type co-infections were observed in 7/86 (8 %) women in CH group, {(HPV56, 58); (HPV58, 43); (HPV58, 42); (HPV 35, 42); (HPV 52, 42, 66, 39); (HPV 52, 43, 66, 39); (HP V56, 33, 42, 39, 68)}. The HPV56 (9/86 = 10 %) is more frequent than HPV58 (6/86 = 7 %) and HPV42 (6/86 = 7 %) followed by HPV 52 (4/86 = 5 %), HPV43(4/86 = 5 %) and HPV 39 (3/86 = 3 %), HPV 45 (2/86 = 2 %), HPV 66 (2/86 = 2 %), HPV35 (1/86 = 1 %), HPV 33(1/86 = 1 %) and HPV 31(1/86 = 1 %) as shown in Fig. 1. Unpredictably, HPV 18 and HPV16 were not detected in CH group. Overall, the co-infection HR-HPV/HIV is present in 31 % of women in CH group while HPV counts including multiple HPV infections were 43.

### HR-HPV status in non-HIV population (group ME)

**Fig. 2:**
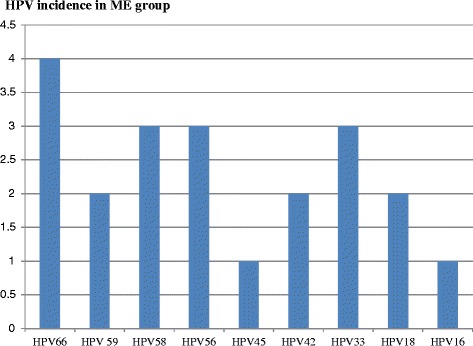
Incidence of HPV type in women without HIV infection in group ME. Genotype HPV 66 is more frequent than HPV58, HPV56, HPV33 followed by HPV59, HPV18 and HPV45, HPV16

Dual and triple HR-HPV co-infections were observed in 5/86 (5 %) of women in group ME: [HPV 45, 56]; [HPV43, 44]; [HPV18, 66]; [HPV42, 59, 68]; [HPV42, 59, 39]. In group ME, HPV 66 (4/86 = 5 %) is more frequent than HPV58 (3/86 = 3 %), HPV56 (3/86 = 3 %), HPV33 (3/86 = 3 %) followed by HPV59 (2/86 = 2 %), HPV18 (2/86 = 2 %) and HPV45 (1/86 = 1 %), HPV16 (1/86 = 1 %) as shown in Fig. 2. Overall, HR-HPV infection was observed in 23 % women of ME group while total HR-HPV counts including multiple HR-HPV infections were 27. The diversity of HR-HPV genotypes makes hard to use HPV as biomarker to follow women toward cancer development in both groups.

### Analysis of HR-HPV prevalence in HIV and non-HIV populations (group CH vs group ME)

**Fig. 3:**
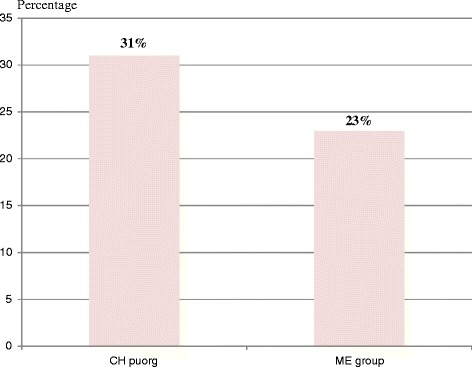
Histogram representing the frequency of women infected with HPV in CH group with HIV and ME group without HIV. The percentage of women with HPV infection in CH group (31 %) is significantly higher than in ME group without (23 %) with *p* < 0.001

More women in CH group have significantly higher HR-HPV infections (27/86 = 31 %) than women in ME group (20/86 = 23 %); *p* < 0.001, as shows in Fig. 3 and Table 1. HR-HPV counts is higher in CH group with HIV infections (*n* = 43) compared to ME group without HIV (*n* = 27), *p* < 0.001. The length of ARV treatment in CH group varies between 1 to 15 years (Table 1).

**Table 1:** Summary of ARV treatment, lamin A/C, HPV and cervical lesions in CH and ME groups

CH group				ME group			
Lamin A/C (*n* = 86)	Women with HPVs	Cervical lesions	Years with ARV (y)	Lamin A/C	Women with HPVs	Cervical lesions	Years with ARV
Normal *N* = 34	9 (10 %)	0	1–13 years	Normal *N* = 35	5 (6 %)	0	No
Low *N* = 22	7 (8 %)	1	1–9 years	Low *N* = 21	3 (3 %)	0	No
Absent *N* = 30	11 (13 %)	3	1–15 years	absent *N* = 30	12 (14 %)	1	No
Total *N* = 86	31 %	5	1–15 years	*N* = 86	23 %	1	No

Table representing the frequency of HPV infection in antiretroviral (ARV) treated women (CH group) and non HIV infected control (ME group). The HPV frequency does not correlate with cervical lesions

### Cervical dysplasia in the context of HIV, HR-HPV and lamin A/C deficiency

**Fig. 4:**
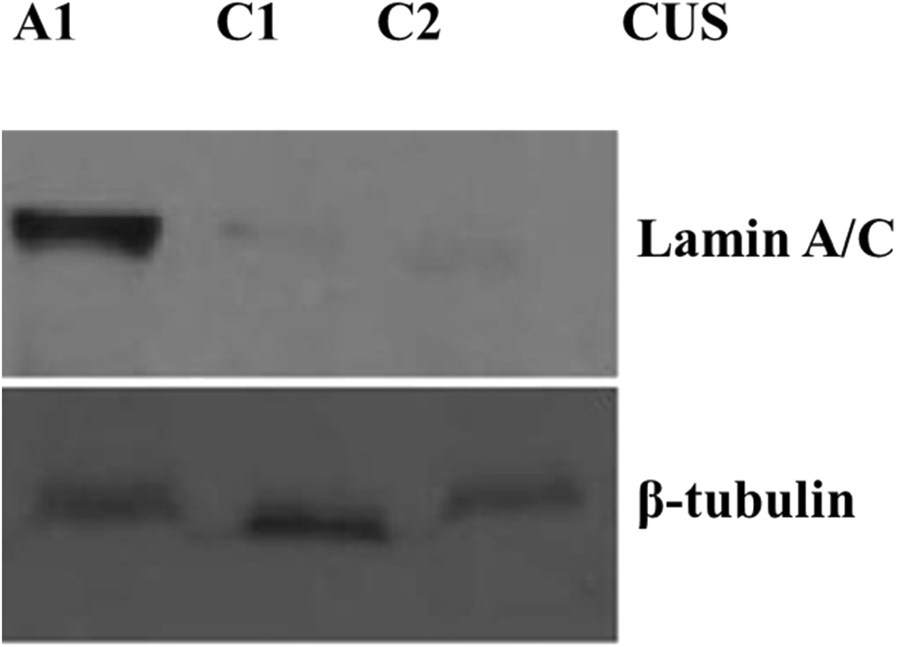
Silencing of Lamin A/C in cervical uterine smears (CUS). An example of western blot showing lamin A/C in CUS samples of women in CH group. Sample A1 expresses of lamin A/C while samples C1 and C2 have lost the expression of lamin A/C

**Fig. 5:**
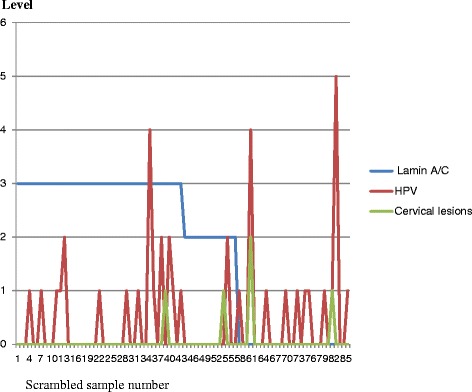
Histogram integrating HPV, lamin A/C and cervical lesions data in CH group infected with HIV. In this CH group cervical lesions are more associated to low or absence of lamin A/C than it is associated to HPV infection

**Fig. 6:**
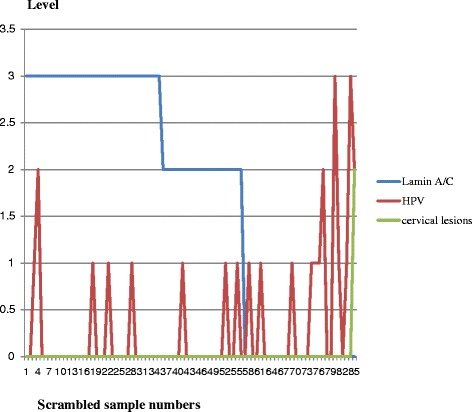
Histogram integrating HPV, lamin A/C and cervical lesions data in ME group without HIV infection. This ME group has less cervical lesions associated to low or absence of lamin A/C

The prevalence of cervical dysplasia in both groups is significantly lower than the prevalence of HR-HPV infections and it is opportune in this instance to evaluate the risk to develop cervical dysplasia with the deficiency in lamin A/C. An example of western blot performed with cell lysates derived from CUS samples in CH group is shown in Fig. 4 with samples expressing lamin A/C and without lamin A/C. Sample A1 expresses lamin A/C while samples C1 and C2 have totally lost the expression of lamin A/C. Samples C1 and C2 have more risk to develop cervical cancer regardless of HIV and HR-HPV status. Multiple HR-HPV co-infections were most observed in women with low lamin A/C or no lamin A/C along with cervical lesions (Figs. 5 and 6).

In CH group cervical dysplasia was observed in 4/86 (5 %) of women living with HIV. We observed low grade cervical intraepithelial lesions (CIN-1) in one woman (52 years old) with co-infections HIV/HPV58 and HPV43; high grade lesions (CIN-2) in one woman (49 years old) with co-infections HIV/HPV52, HPV43, HPV 66 and HPV39; condylomatous epithelial lesions in one woman (26 years old) with co-infections HIV/HPV56, HPV33, HPV42, HPV39 and HPV68; condylomatous epithelial lesions in one woman (28 years old) with co-infections HIV/HPV HPV35 and HPV42. One woman with co-infections HIV/HPV52, HPV42, HPV66 and HPV39 has not developed cervical lesions at the time of CUS collection as shown in Fig. 5. The silencing of lamin A/C is observed in 34 % of group CH and this put them at risk to develop cervical dysplasia in the future. All 4 women with cervical lesions in CH group have lost the expression of lamin A/C at the time of CUS collections associated with co-infection HIV/multiple HR-HPVs. Conversely not all HIV/multiple HR-HPV co-infections developed cervical lesions at the time of sample collection. The histogram integrating lamin A/C, HR-HPV and cervical lesions data in CH group infected with HIV is shown in Fig. 5 and verified that cervical lesions are more associated to the deficiency of lamin A/C than to HR-HPV infection.

In ME group without HIV infection, one woman with HPV18 has developed cervical dysplasia (CIN I) at the time of sample collection (1/86 or 1 %) associated to the loss of lamin A/C. All other women with HPV infection have not developed cervical lesions at the time of sample collection but the silencing of lamin A/C is observed in 34 % of them and this put them at risk to develop cervical dysplasia in the future (Fig. 6). Table 1 represents the prevalence of HR-HPV infection among antiretroviral (ARV) treated women (CH group) and non HIV infected control (ME group) and shows that HR-HPV infection is not always associated with cervical lesions.

When considering the subgroup with lamin A/C deficiency, the prevalence of HR-HPV infection in CH group is 13 % while the prevalence of HR-HPV infection in ME group is14% as shown in table; the difference was not significant. In both groups the absence of lamin A/C is a suitable prognostic factor for the risk to develop cervical cancer in the future.

### Two years follow-up of the women participating in this study

**Fig. 7:**
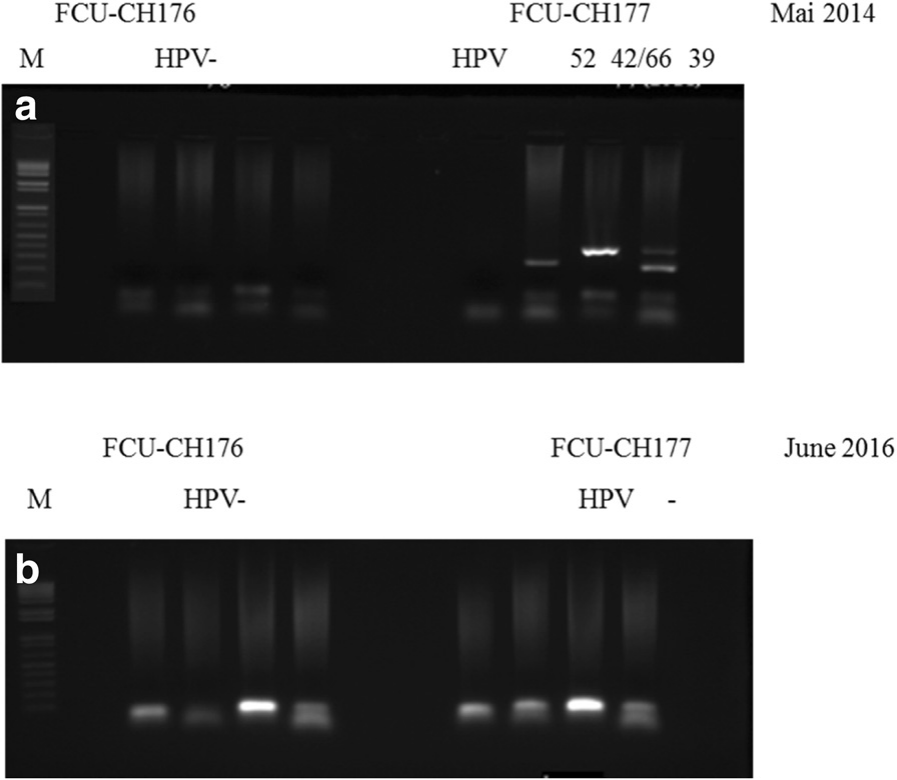
Two years follow-up of HPV infected patients. **a** Agarose gel with HPV PCR products showing HPV negative sample CUS FCU-CH176 and HPV positive sample CUS FCU-CH177 with quadruple HPVs (type 52, 42, 66, 39) in Mai 2014. **b** HPV negative sample CUS FCU-CH176 and sample CUS FCU-CH177 without HPV 2 years later in June 2016. Sample numbers are scrambled and could not be traced to the patients

Both groups are under the supervision of gynecologists and are advised to perform CUS or colposcopy once a year. We recalled all women investigated after our first analysis in Mai 2014 for a 2 years follow-up in June 2016. Among the CH group, no new cases of cervical dysplasia were reported; unexpectedly the woman with co-infections HIV/HPV52, HPV42, HPV66 and HPV39 with no cervical lesions at the time of CUS collection did not develop cervical lesions 2 years later but did clear out all HPV genotypes as shown by gel photo in Fig. 7 (CUS FCU-177) along with negative control (CUS FCU-176). The 26 years old woman with quintuple HR-HPV (HPV56, HPV33, HPV42, HPV39 and HPV68) and condylomatous lesions is still receiving ARV treatment and is in good health. All other women are still receiving ARV treatment for free and are in good health. Among the ME group, one woman with HPV and no lamin A/C developed cervical lesions and two women with no HPV but with total absence of lamin A/C developed cervical lesions and were treated by the gynecologist. Sample numbers are scrambled and could not be traced to the patients.

## Discussion

HR-HPV genotypes fluctuate in each country and environment as well as the association of HIV/HR-HPV co-infections to cervical dysplasia or cervical cancer [1, 3, 12]. Our study in the south of BENIN shows that HPV56 is predominant among women leaving with HIV and is more frequent than HPV 58 and HPV42 and HPV 66. Among women in control ME group with no HIV infection, the HPV 66 is predominant and is more frequent than HPV58, HPV56.

In study done in Democratic Republic of the Congo (Kinshasa) the most common HR-HPV types among HIV-positive women were HPV68, HPV35, HPV52 and HPV16. Among women with negative/unknown HIV status, the most common HR-HPV types were HPV52, HPV35 and HPV18 as reported [1]. In southwest China (Yunnan) the most common HR-HPV types in women infected with HIV were HPV52, HPV58, HPV18, HPV16, and HPV33 as reported [4]. In Thailand the most common HR-HPV types in the population were HPV72, HPV52, HPV62, and HPV16 [3].

Although HR-HPV infections are more frequent in CH group with HIV (Fig. 4), not all women infected with HR-HPV will develop cervical dysplasia or cancer (Figs. 5 and 6) and it is opportune to integrate other cancer biomarkers with HR-HPV analyses to efficiently predict the risk to develop cervical dysplasia independently to viral infection. The discrepancy between the low rate of cervical intra epithelial lesions and the high rate of HR-HPV infections is striking in CH group and is an indicator that the evaluation of HR-HPV genotypes solely is not sufficient to define the risk to develop cervical lesions in women living with HIV and under efficient ARV treatment. The early detection of cervical lesions in women with HIV allowed the gynecologist to apply cryotherapy for these women.

Aside of viral integration into the genome, nuclear protein anomalies and chromosomal instabilities occur to initiate tumor formation and progression [14–17]. The deficiency of lamin A/C was already demonstrated in several studies to be a reliable prognostic factor for cervical, ovarian, breast, prostate, gastro-intestinal, colon cancers and neuroblastoma [11, 12, 14–21]. In BENIN, cervical cancer ranked number one among women infected with HIV but appreciative to the efficacy of ARV treatments received in CH group the prevalence of cervical dysplasia is not enormous as predicted by studies based on HR-HPV infection rate [8, 13]. HPV did clear out from some women without triggering cervical lesions as shown reported by our 2-year follow-up periods.

Although the prevalence of HPV infection is significantly higher in the CH group, the frequency of HR-HPV infection adjusted to the deficiency of lamin A/C is not significantly different in both groups investigated making this assessment more predictable of the risk to develop cervical cancer in the future. Due to the diversity of virus types involved in cervical cancer development and progression, we recommend to investigate further more molecular biomarkers in living individual for the screening and prevention of cervical cancer rather than taking solely into consideration HR-HPV infection rate especially in African countries.

## Conclusion

Women living with HIV are more sensitive to multiple HR-HPV infections but not all HR-HPV infections generate cervical dysplasia. The effectiveness of antiretroviral therapy in CH group may reduce significantly the frequency of cervical dysplasia although it has no influence on the prevalence of HR-HPV/HIV co-infection. Lamin A/C should always be used as biomarker for cervical cancer screening associated to HR-HPV genotypes in African countries to not skip women who develop cervical dysplasia independently of HR-HPV infections.

## Abbreviations

ARV, Anti-retroviral; CUS, cervical uterine smears; EDTA, Ethylenediaminetetraacetic acid (EDTA); HIV, Human immunodeficiency Virus; HPV, Human Papilloma Virus; HR-HPV, high risk Human Papilloma Virus; HRP, Horseradish Peroxidase; KCl, potassium chloride; NaCl, Sodium chloride; NaF, sodium fluoride; NaN_3_, Sodium azide; PBS, phosphate Buffered Saline; PMSF, Phenyl-Methyl-Sulfonyl Fluoride; SDS, sodium dodecyl sulfate; TBS, Tris Buffer Saline; TBST, Tris Buffer Saline plus Tween-20; Tris-HCl, Tris-Hydrochloride
